# Tau pathology induces loss of GABAergic interneurons leading to altered synaptic plasticity and behavioral impairments

**DOI:** 10.1186/2051-5960-1-34

**Published:** 2013-07-11

**Authors:** Josien Levenga, Pavan Krishnamurthy, Hameetha Rajamohamedsait, Helen Wong, Thomas F Franke, Peter Cain, Einar M Sigurdsson, Charles A Hoeffer

**Affiliations:** 1 Department of Physiology & Neuroscience, New York University School of Medicine, 550 First Ave, SRB 610, New York, NY 10016, USA; 2 Druckenmiller Neuroscience Institute, New York University School of Medicine, 550 First Ave, SRB 610, New York, NY 10016, USA; 3 Department of Psychiatry, New York University School of Medicine, 550 First Ave, MSB 459, New York, NY 10016, USA; 4 Department of Biochemistry and Molecular Pharmacology, New York University, School of Medicine, New York, NY 10016, USA; 5 Department of Psychology, Excelsior College, Albany, NY 12203, USA

**Keywords:** Tau, P301L, Synaptic plasticity, Behavior, Hippocampus, GABAergic interneurons

## Abstract

**Background:**

Tau is a microtubule stabilizing protein and is mainly expressed in neurons. Tau aggregation into oligomers and tangles is considered an important pathological event in tauopathies, such as frontotemporal dementia (FTD) and Alzheimer’s disease (AD). Tauopathies are also associated with deficits in synaptic plasticity such as long-term potentiation (LTP), but the specific role of tau in the manifestation of these deficiencies is not well-understood. We examined long lasting forms of synaptic plasticity in JNPL3 (BL6) mice expressing mutant tau that is identified in some inherited FTDs.

**Results:**

We found that aged (>12 months) JNPL3 (BL6) mice exhibit enhanced hippocampal late-phase (L-LTP), while young JNPL3 (BL6) mice (age 6 months) displayed normal L-LTP. This enhanced L-LTP in aged JNPL3 (BL6) mice was rescued with the GABA_A_R agonist, zolpidem, suggesting a loss of GABAergic function. Indeed, we found that mutant mice displayed a reduction in hippocampal GABAergic interneurons. Finally, we also found that expression of mutant tau led to severe sensorimotor-gating and hippocampus-dependent memory deficits in the aged JNPL3 (BL6) mice.

**Conclusions:**

We show for the first time that hippocampal GABAergic function is impaired by pathological tau protein, leading to altered synaptic plasticity and severe memory deficits. Increased understanding of the molecular mechanisms underlying the synaptic failure in AD and FTD is critical to identifying targets for therapies to restore cognitive deficiencies associated with tauopathies.

## Introduction

Tau is a microtubule binding protein, primarily expressed in the central nervous system [1]. It is predominantly localized to axons, regulating the stability of axonal microtubules [2,3]. Tau has also been identified in dendrites, albeit at lower levels than in axons [4]. Tau is highly soluble and its activity is regulated by phosphorylation at specific sites (reviewed in [5]). In frontotemporal dementia (FTD) and Alzheimer’s disease (AD), tau becomes hyperphosphorylated, leading to an accumulation of tau in the somatodendritic compartment that eventually forms a neurofibrillary tangle (NFT). NFTs are thought to lead to neuronal dysfunction and neuronal death [5]. However, some recent evidence shows that NFTs are not toxic *per se*, but instead soluble hyperphosphorylated tau protein, present in early and late stages of disease, are the key pathogenic species [6]. In both scenarios, tauopathy leads to a loss of synapses and neurons with associated cognitive and behavioral impairments [7,8].

Synaptic impairments in several AD mouse models that express mutant amyloid precursor protein (APP) [9-11] and hippocampal slices treated with Aβ oligomers [12,13] have established the importance of Aβ pathology in synaptic function in AD. Tau has been proposed as a key mediator of Aβ-associated cellular and cognitive defects [14,15] and Aβ-directed long-term potentiation (LTP) deficits [16]. These findings may indicate that tau lesions promote synaptic deficits independent of Aβ-directed effects. In support of this notion, a few pure tauopathy models have been associated with aberrant LTP [17-19]. However, these studies examined very transient forms of LTP and examined plasticity in young mice, before the onset of tau pathology. Compared to the significant effort made exploring Aβ-mediated effects on synaptic function, much less is understood about the role of pathological tau protein in synaptic deficits.

Recent studies have shown that neural network abnormalities in the form of circuit hyperexcitability either precede or lead to AD. For example, the incidence of epilepsy is increased in patients with AD, suggesting an imbalance in excitation and inhibition [20-22]. Also, in patients with AD and in aged individuals, amyloid plaques are found distributed along networks displaying abnormal activity [23,24]. Finally, tau might play an important role in circuit hyperexcitability, since it was found that mutant P301L tau expression resulted in electrophysiological changes in cortical pyramidal neurons [25]. These changes include a depolarized resting membrane potential, increased depolarizing sag potential and increased action potential firing rates, which are all indications of hyperexcitability. Taken together, these findings strongly support the notion that neuronal network balance is disturbed in tauopathies, and one possible mechanism that may lead to this imbalance is impairment of inhibitory neurotransmission.

Inhibitory neurotransmission in the brain is largely mediated by γ-aminobutyric acid (GABA) acting through GABA type A receptors (GABA_A_R)s and GABA type B receptors (GABA_B_R)s. Changes in GABAergic transmission are implicated in the regulation of all aspects of brain function, since they are critical for maintaining the proper balance of activity in the brain. Deficits in GABA_A_R-mediated transmission are implicated in the etiology of epilepsy [26], anxiety [27], mood disorders [28], aging, and AD [29]. Loss of normal excitatory/inhibitory balance resulting from dysregulation of GABAergic signaling may underlie increased incidence of epileptic seizures in AD patients [30]. These data suggest that AD-related cognitive impairments are likely to be affected by GABAergic dysregulation.

In the present study we examined hippocampus-dependent synaptic plasticity and behavior in aged JNPL3 (BL6) mice, a transgenic (Tg) mouse model expressing human mutant P301L tau in a C57BL6 background. Using several experimental approaches of electrophysiology, histology and behavior modeling, we found that the expression of pathological tau led to significant physiological alterations and behavioral abnormalities in aged JNPL3 (BL6) mice. We found a loss of GABAergic interneurons leading to electrophysiological alterations, sensorimotor deficits, and severe hippocampus-dependent memory deficits.

This work has the potential to provide valuable translational insight into AD treatments by validating how strategies for GABAergic manipulation and tau immunoclearance may restore synaptic function in the AD brain.

## Materials and methods

### Animals

JNPL3 (BL6) transgenic mice (Taconic, New York) were backcrossed in a C57BL/6 background (>10 generations). Age-matched control wild-type (WT) mice were purchased from Taconic. Mice were maintained on a 12:12 hour L:D schedule with food and water available *ad libitum*. Mice were tested at six, 12 or 18 months of age, depending on the experiments. Procedures were approved by the New York University School of Medicine Institutional Animal Care and Use Committee.

### Brain fractionation protocol for Western blot analysis

Tau solubility was analyzed using a modified protocol from [31-33]. Briefly frozen cortex sections were homogenized without thawing in 5 X vol/wg of RIPA buffer (50 mM Tris–HCl, pH 7.4; 1% Nonidet P-40; 0.25% Na-deoxycholate; 150 mM NaCl; 1 mM EDTA; 1 mM PMSF; 1 mM Na_3_VO_4_; 1 mM NaF; Complete protease inhibitor cocktail, Roche, IN, USA), with a mechanical homogenizer (TH; Omni International, USA), and centrifuged at 20,000 X g for 20 min at 4°C. An aliquot of the supernatant representing the total tau fraction was kept for protein quantification and western blot analysis. The rest of the supernatant was adjusted to 1% sarkosyl (N-lauroylsarcosine), incubated for 30 min at room temperature with constant rotating, and centrifuged at 100,000g for one hr at 20°C. After high speed centrifugation, the pellet was washed with 1% sarkosyl and centrifuged again at 100,000g for one h at 20°C. The post wash pellet containing sarkosyl-insoluble, aggregated tau was resuspended and analyzed by SDS-PAGE. Tau in the sarkosyl pellet has been shown by immuno-electron microscopy to be filamentous [32], and it is synonymous with that identified by immunohistochemistry in NFTs. All fractions were diluted in O+ buffer (62.5 mM Tris–HCl, pH 6.8; 10% glycerol; 5% 2-mercaptoethanol; 2.3% SDS; 1 mM EGTA; 1 mM EDTA; 1 mM PMSF; 1 mM Na_3_VO_4_; 1 mM NaF; Complete protease inhibitor cocktail, Roche), a modified O buffer, boiled for 5 min, and kept at −20°C. Depending on the antibody used, 10 to 20 μg of protein were analyzed by western blotting. Equal amount of protein (BCA assay, Promega) was loaded and the samples were electrophoresed on 10-12% SDS-PAGE gels and transferred to nitrocellulose membranes. All blots were blocked (5% nonfat milk and 0.1% Tween 20 in TBS) and then incubated with various primary antibodies overnight. Subsequently, the blots were washed and incubated for 2 h at room temperature with peroxidase-conjugated, goat anti-rabbit (Thermo Scientific) or anti-mouse IgG (1:2000; Jackson ImmunoResearch). Immunoreactive bands were visualized and analyzed by enhanced chemiluminescent reagent (Pierce ECL, Thermo Scientific) using a Fujifilm LAS4000 imaging system and the Multi Gauge software (Fujifilm Life Science). To compare the relative amount of tau protein, the densities of the immuno-reactive bands corresponding to phospho-tau were normalized and reported relative to the amounts of total tau protein or α-tubulin.

### Electrophysiology

Transverse hippocampal slices (400 μm) were prepared from one hemisphere of age-matched mice (12–18 months or 6–7 months of age) using a vibratome (from part of the cohort, the other hemisphere was used to perform immunostaining or in situ hybridization, see below). Slices were maintained in oxygenated ACSF containing the following (in mM): 125 NaCl, 2.5 KCl, 1.25 NaH_2_PO_4_, 25 NaHCO_3_, 25 D-glucose, 2 CaCl_2_, and 1 MgCl_2_ at room temperature. For electrophysiology experiments, slices were transferred to interface recording chambers (preheated to 30°C) perfused with oxygenated ACSF. Extracellular field EPSPs (fEPSPs) were evoked by stimulation of Schaffer collateral pathway afferents and were measured by recording in stratum radiatum of area CA1. In order to determine the response range for each hippocampal slice, the stimulus range was divided into 10 arbitrary units. The slices were stimulated at each level, and the fEPSP was recorded. In each recording, the fiber volley amplitude was as a measure of the input stimulation to the fEPSP. The range of input values and their respective output values (measured as the fEPSP slope) were plotted as a mean to characterize basal synaptic transmission in transgenic and wild-type mice across all experiments. Baseline responses were calculated using the stimulation intensity that elicited 40-50% of the maximal fEPSP response as determined by the input–output relationship. Paired-pulse facilitation (PPF), an assay of normal presynaptic function, was induced with two stimuli of equal intensity (same as baseline intensity) presented in rapid succession at variable interpulse intervals, ranging from 10 ms to 300 ms. PPF was measured by examining the ratio of the fEPSP slope in response to stimulus 2 and that of stimulus 1. Before LTP-inducing high-frequency stimulation (HFS), stable baseline synaptic transmission was established for 20–30 min with a stimulus intensity of 40–50% of the maximum fEPSP. Stimulus intensity of the HFS was matched to the intensity used in the baseline recordings. LTP was induced by either one or four trains (2 min intertrain interval) of 100 Hz HFS for 1 s. Data were collected and presented as the average slope of the fEPSP from six individual traces collected over 2 min and then normalized to baseline recordings of fEPSPs. Hippocampal slices from Tg and WT mice were prepared simultaneously and placed in a chamber outfitted with dual-recording equipment, thereby minimizing day-to-day variability in slice preparations and recordings. For zolpidem treatment, slices were incubated 20 min prior to HFS with a subthreshold concentration of zolpidem (1 μM). Student’s t-test, Repeated Measures ANOVA or N-way ANOVA (where appropriate) were used for electrophysiological data analysis with p < 0.05 as significance criteria.

### In situ hybridization

Brains (one hemisphere from mice also used for electrophysiology) were fixed overnight in buffered 2% paraformaldehyde (PFA) at 4°C. Next day, hemispheres were stored in 20% glycerol, 2% DMSO in phosphate buffer (PB) until the brains were sliced as consecutive serial coronal sections (5 series, 50 μm thickness). Brain sections were stored in cryoprotectant (300 ml ethylene glycol, 550 ml PB, 300 g Sucrose, volume to 1000 ml with H_2_O; pH7.2) until used. Serial brain sections were used to perform *in situ* hybridizations as previously described in [34]. Briefly, RNA probes were prepared using dioxygenin (DIG) RNA labeling kits (Roche). Sections were postfixed in 4% PFA for 10 min followed by a wash in phosphate-buffered saline (PBS). Sections were treated with 1.5% H_2_O_2_ in methanol and rinsed in PBS. Then, sections were treated with 0.2 M HCl and washed in PBS. Proteinase K (Roche) digestion (20 μg/mL in PBS) was carried out followed by a wash in PBS, and the sections were refixed for 5 min in 4% PFA and washed with PBS. The sections were acetylated for 10 min (2.2 g triethanolamine hydrochloride (Sigma), 540 μL of 10 N NaOH (Fisher Scientific), 300 μL of acetic anhydride (Sigma) in 60 mL water) and washed in PBS. RNA probes, prepared at a dilution of 2 μL/mL in hybridization solution (50% formamide, 10% dextran sulfate, 1% 100× Denhart's, 250 μg/mL yeast tRNA, 0.3 M NaCl, 20 mM Tris–HCl, pH8, 5 mM EDTA, 10 mM NaPO4, 1% sarkosyl), were incubated at 80°C for 2 min. Thereafter, 500 μL of the probe mix was applied to the brain sections and incubated at 55°C overnight. The next day, sections were subjected to high stringency wash in pre-warmed 50% formamide, 2× SSC at 65°C. Next, the sections were rinsed in RNase buffer (0.5 M NaCl, 10 mM Tris–HCl, pH 7.5, 5 mM EDTA), followed by an RNaseA (Roche) treatment (20 μg/mL in RNase buffer) for 30 min and followed by a wash in RNase buffer, all at 37°C. The high stringency washes were repeated twice for 20 min each at 65°C, followed by a 15 min rinse in 2× SSC, then 0.1× SSC, both at 37°C. Sections were then washed in Wash Buffer (WB, 100 mM maleic acid, 150 mM NaCl, 0.5% Tween-20) and blocked with Blocking Buffer (1% Boehringer Manheim in WB) followed by incubation with anti-DIG-POD antibody (Roche) overnight at 4°C. Next day, slices were washed in WB and then incubated with Blocking Buffer 2 (0.5% Casein, 150 mM NaCl, 100 mM Tris, pH 7.5). Next biotinyl-tyramide was added to the sections, followed by washes in WB. Then sections were incubated with Streptavidin – AP for 1h at room temperature followed by 3 washes in WB and a wash in NTMT buffer (100 mM NaCl, 100 mM Tris–HCl, pH 9.5, 50 mM MgCl_2_, 0.1% Tween-20). The sections were then placed in a light-protected environment with approximately 400 μL of BM-purple AP substrate (Roche) until satisfactory staining was achieved. Finally, the sections were rinsed twice in PBS, coverslipped using Crystal mount aqueous mounting media (Sigma) and images were acquired using a Leica DM 5000B light microscope. Using Image J software (National Institutes of Health), regions of interests (ROI) were defined in all consecutive slices of one mouse. The size of ROI varied to some extent from anterior to posterior based on each individual slice. General criteria for the CA1 ROI were determined by boundaries starting at the CA2-CA1 border till the subiculum-CA1 boundary, including the stratum oriens and stratum radiatum. Within these ROIs, the number of positive cells was counted. The number of positive cells was corrected to the volume of the ROIs. All cell counts were made by observers blind to genotype.

### Immunohistochemistry

For fluorescent immunostaining to count number of SST or PV positive cells from mice used for electrophysiology, consecutive serial brain sections (50 μm thick) of freshly fixed brain stored in cryoprotectant (see above), were washed in PBS for 3× 10 min. For blocking and permeabilization we used “staining buffer” containing 0.05 M Tris, 0.9% NaCl, 0.25% gelatin, and 0.5% Triton X-100, pH 7.4. Primary antibodies, rabbit anti-somatostatin (1:1000; Peninsula Laboratories/Bachem) and mouse anti-parvalbumin (1:500; Sigma-Aldrich) were diluted in staining buffer and incubated overnight at 4°C. The next day, the brain sections were washed in PBS and incubated with donkey anti-mouse Cy3 antibody (1:200; Jackson Immunoresearch) and donkey anti-rabbit Alexa 488 antibody (1:200; Jackson Immunoresearch), or with donkey anti-mouse Alexa 488 (1:200; Jackson Immunoresearch) and donkey anti-rabbit Cy3 antibody (1:200; Jackson Immunoresearch) diluted in staining buffer with Hoechst for one h at room temperature. Finally, the brain slices were washed in PBS and mounted in Mowiol mounting solution (Mowiol 4–88). In order to examine if pathological tau was expressed in GABAergic interneurons, two other aged JNPL3 (BL6) mice (age 13 months) were perfused with 0.9% saline, followed by perfusion of 4% paraformaldehyde (PFA) in PBS. Brains were fixed overnight in buffered 2% paraformaldehyde at 4°C. Next day, hemispheres were stored in 20% glycerol, 2% DMSO in phosphate buffer (PB) until the brains were sliced as consecutive serial coronal sections (5 series, 50 μm thickness). Brains slices were immunostained as described above and incubated with rabbit anti-somatostatin (1:1000; Peninsula Laboratories/Bachem) or rabbit anti-parvalbumin (1:1000, Swant) and with mouse anti-PHF1 (1:500; gift from Dr. Peter Davies) or mouse anti-MC1 (1:100; gift from Dr. Peter Davies). Images of brains were acquired using a Zeiss LSM510 confocal microscope. To quantify the number of GABAergic interneurons per hippocampal region, the complete hippocampus was imaged using Zeiss Zen2009 software. Next, Z-stacked images were stacked to a maximum intensity projections and ROIs were defined in all consecutive slices of one mouse, i.e. hippocampal CA1 and dentate gyrus (DG) in Image J. ROI varied from posterior to anterior. General criteria for the DG ROI were determined by the boundaries of the granular layers. Criteria for the CA1 ROIs were determined as described above. Within these ROIs, the number of positive cells was counted. The average of all positive cells was corrected to the volume of the ROIs.

For DAB staining, consecutive serial brain sections (50 μm thickness) stored in cryoprotectant, were washed in TBS (10 mM Tris, 140 mM NaCl, pH 7.4), then incubated with 3% H_2_O_2_/0.25% Triton X-100 (Sigma) for 30 min and followed by incubation in 5% milk in TBS. Brain slices were then incubated overnight with mouse anti-PHF1 (1:1000) or mouse anti-MC1 (1:100) in 5% milk in TBS. Slices were then washed in TBS +0.05% Triton-X100 (TBS-T), incubated with goat anti-mouse-Biotin (1:1000; M.O.M. kit, Vector laboratories) secondary antibody diluted in 20% Superblock (Pierce)/TBS-T for 2 hours, washed with TBS-T, and incubated with ABC reaction (M.O.M. kit) in Superblock in TBS-T. Brain slices were washed in 0.2 M sodium acetate and developed in DAB + nickel ammonium sulfate (17mg of DAB, 1.25 g Nickel ammonium sulfate in 50 ml of 0.2 M sodium acetate and 0.3% H_2_O_2_). Slices were washed in sodium acetate and TBS and mounted. Images of brains were acquired using a Leica DM 5000B light microscope.

### Biotin surface labeling

Biotin surface labeling was performed as described in [35]. Briefly, hippocampal slices were generated as described for electrophysiology. Slices were maintained in oxygenated ACSF containing the following (in mM): 125 NaCl, 2.5 KCl, 1.25 NaH_2_PO_4_, 25 NaHCO_3_, 25 D-glucose, 2 CaCl_2_, and 1 MgCl_2_ at 32°C for at least 1 h to recover. Then slices were treated with 0.5 mg/ml Sulfo-NHS-SS-biotin (Pierce) for 45 min on ice to label surface proteins. Slices were then washed in Tris-ACSF (25mM Tris pH 7.2 + ACSF). Slices were snap frozen and lysed in homogenization buffer (in mM: 40 HEPES pH 7.5, 150 NaCl, 10 pyrophosphate, 10 glycerophosphate, 1 EDTA) containing protease inhibitor and phosphatase inhibitor cocktail II and III (Sigma). Homogenates were cleared by centrifugation at 13000 rpm for 10 min at 4°C. Then samples were run on a SDS-page gel and immunoblotted against GABA_A_R alpha1 (1:1000, Neuromab), GABA_A_beta2/beta3 (1:1000, Upstate) and GAPDH (1:10000, Cell Signaling).

### Behavioral studies

#### Contextual fear conditioning

Tg and control mice were tested at the age of 5–6 or 12–13 months. Apparatus: Mice were trained and tested using the FreezeFrame system (Coulbourn Instruments). For training, mouse test cages equipped with stainless-steel shocking grids were connected to a precision feedback current-regulated shocker (Coulbourn Instruments). Each test cage was contained in a sound-attenuating enclosure (Coulbourn Instruments). Behavior was recorded using low-light video cameras. Stimulus presentation was automated using Actimetrics FreezeFrame software version 2.2 (Coulbourn Instruments). All equipment was thoroughly cleaned with water followed by isopropanol between sessions. Fear conditioning: Mice were habituated for 2 min on a shocking grid (context: shocking floor grids, vanilla scent). Fear conditioning was conducted with three 2 s, 0.5-mA footshocks (US) separated by 30 s. After conditioning, mice were returned to their home cages. Fear memory: Mice were retested in the training context (shocking grid, vanilla scent) 1 h 24 h and 7 days after training. Freezing behavior was measured using FreezeFrame (Coulbourn Instruments). Student’s t-test was used to analyze the data with p<0.05 as significant criteria.

#### Prepulse inhibition (PPI)

PPI test was used to study sensorimotor gating. Tg and control mice were tested at the age of 5–6 or 12–13 months. The testing apparatus of a startle response system was contained in a sound attenuating chamber calibrated for responses from mice that are 20–35 g in weight (San Diego Instruments, San Diego, USA). Each mouse was placed in a clear, cylindrical holding tube within a sound-attenuating chamber and habituated for 4 min immediately prior to testing. The test started with 5 startle pulses of 120 dB to measure startle responses. This was followed by 5 blocks of randomized trials: no stimulus, startle stimulus (120 dB), 72 dB prepulse+120 dB startle, 84 dB prepulse+120 dB startle, 90 dB prepulse+120 dB startle. The test was finalized by 5 startle pulses of 120 dB to measure habituation. The prepulses were presented 100 ms before startle stimulus. The inter-block interval ranged from 6–20 s. All tests were performed at the same time of day following identical habituation periods. Mice failing to demonstrate acoustic startle response at 2,5× baseline were excluded as hearing impaired. Responses were detected as changes within the holding tube. Student’s t-test was used to analyze the data with p<0.05 as significant criteria.

#### Hotplate

The hotplate test was used to determine any differences in nociceptive responses. Mice were placed on a hotplate preheated to 50°C (Columbus Instruments). The animal was observed and the time for the animal to lift one of its hind paws was recorded. The mice were then immediately removed from the hotplate and placed back in their cage. The hind paw is used to determine nociception because lifting the front paws is also normal exploratory behavior. Student’s t-test was used to analyze the data with p<0.05 as significant criteria.

## Results

### Tau pathology is evident in aged JNPL3 (BL6) mice

**Figure 1:**
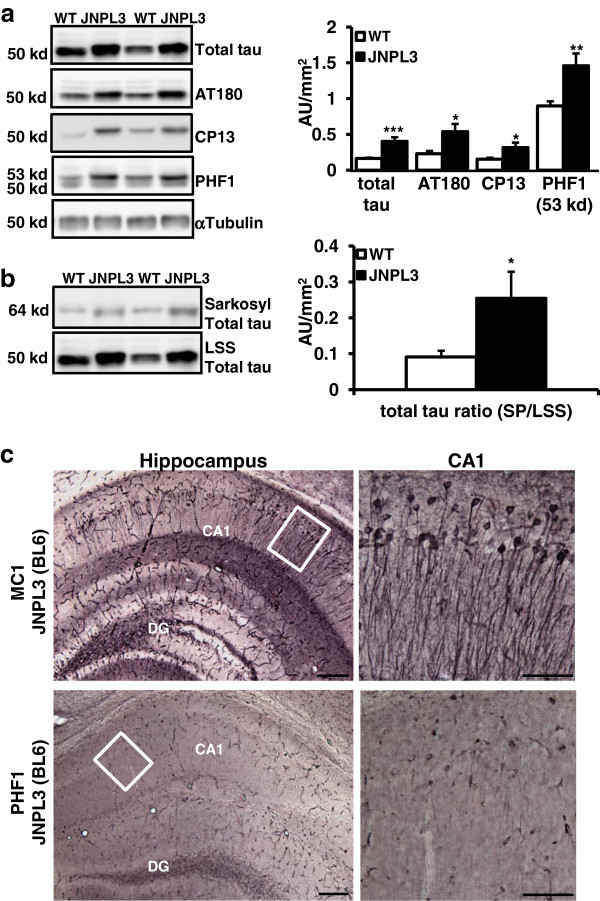
**Aged JNPL3 (BL6) mice show increased tau pathology in the cortex and hippocampus. (a)** Western blot analysis of total tau levels and phospho-tau status in low speed supernatant (LSS) fractions. Total tau levels were significantly increased in Tg mice compared to age-matched WT mice (*n*=14 per group, *t*_26_=4.616, ***P=0.0002). Early-stage pathological phospho-tau markers, AT180 and CP13, were also significantly increased in cortical tissue from Tg mice (AT180: *t*_26_=3.674, *P=0.0011; CP13: *t*_26_=2.156, *P=0.0405). Immunoblotting with the PHF1 antibody showed a significant shift in the banding pattern from 50 kd to 53 kd in Tg mice when compared to WT controls (*t*_26_=2.527, *P=0.0179). **(b)** Levels of insoluble sarkosyl pellet tau normalized to LSS total tau are increased in Tg mice compared to controls (*t*_26_=2.215, *P=0.0357). **(c)** Tg mice showed positive immunostaining with pathological-tau MC1 and PHF1 antibodies. Both antibodies showed staining in the CA1 of JNPL3 (BL6) mice, although to a lesser extent for the PHF1 than MC1 antibody. Inset is higher magnification of the highlighted area. Since brains were fixed with paraformaldehyde without perfusing, blood vessel staining was also present (DG=dentate gyrus, CA1= hippocampal area CA1; Scale bar = 200 μm). Data presented as mean + SEM.

JNPL3 mice, originally generated in a mixed genetic background, develop severe pathological phenotypes, such as neurofibrillary tangles [36]. In our study, we used JNPL3 mice in a C57BL/6 background JNPL3 (BL6) (young: 5–6 months or aged: 12–18 months). To determine if markers of pathological tau were present in aged mice, we examined levels of total tau and phosphorylated tau in the cortex of JNPL3 (BL6) and age-matched C57BL/6 wild-type (WT) control mice. We found that the soluble protein fraction (low speed supernatant) derived from Tg mice showed increased total tau levels compared to WT mice (Figure 1a). In addition, early and late-stage pathological phosphorylation sites of tau, recognized by CP13, AT180, and PHF1 antibodies, were significantly increased in the Tg mice (Figure 1a). Tau protein levels in the sarkosyl pellet, which contains insoluble tau aggregates, were also increased in Tg mice (Figure 1b). Next we analyzed brain tissue from aged Tg mice with the pathological-tau antibodies MC1 and PHF1. MC1 immunostaining, an early-stage conformational tau pathology marker, was found throughout the brain of Tg mice, including in the molecular layer of the hippocampus (Figure 1c). PHF1 staining, a late-stage phospho-tau pathology marker, also showed staining in Tg mice but was less abundant than MC1 staining (Figure 1c). In WT mice, no positive cells were identified (Additional file 1: Figure S1). Combined these data clearly demonstrate that tau pathology is present in the aged JNPL3 (BL6) mice used for this study.

### Altered hippocampal LTP in aged JNPL3 (BL6) mice

**Figure 2:**
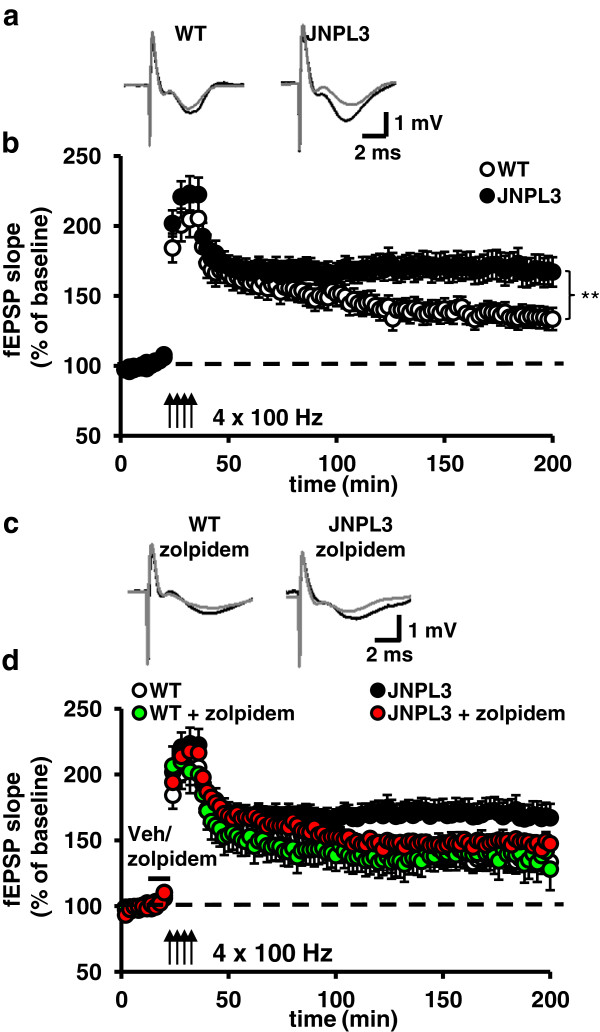
**Aged Tg mice exhibit altered synaptic plasticity which can be rescued with zolpidem, a GABA**_**A**_**R agonist. (a)** Representative traces of field excitatory postsynaptic potentials (fEPSP) in hippocampal slices from aged JNPL3 (BL6) and WT mice that received four trains of high frequency stimulation (HFS) (gray = fEPSP at baseline, black = fEPSP after HFS). **(b)** Late-phase long-term potentiation (L-LTP) is enhanced in aged JNPL3 (BL6) mice compared to WT controls (ANOVA: WT= 9 mice, 24 slices; Tg=12 mice, 28 slices, *F*_*(1,50)*_=7.841, **p=0.007). **(c)** Representative traces of fEPSPs in slices from aged JNPL3 (BL6) and WT mice treated with the GABA_A_R agonist zolpidem and four trains of HFS (gray = fEPSP at baseline, black = fEPSP after HFS). **(d)** Zolpidem (1 μM for 10’) rescued the enhanced L-LTP in Tg mice while having no effect on L-LTP in WT slices (ANOVA: WT zolpidem=6 mice, 11 slices, Tg zolpidem=7 mice, 17 slices, WT veh= 9 mice, 24 slices, Tg veh=9mice, 28 slices, *F*_(1,78)_*=*6.841, *p=0.011. Tukey HSD comparison: Tg zolpidem vs. WT veh: *p*=0.971). Data presented as mean ± SEM.

Having verified tau pathology in aged Tg mice, we next examined synaptic plasticity in the CA3-CA1 circuit in hippocampal slices from Tg and WT animals. Late phase-LTP (L-LTP) was significantly enhanced in aged Tg mice compared to age-matched WT mice (Figure 2a, 2b). An examination of several basal synaptic functions such as input/output (I/O), paired-pulse facilitation (PPF), and early phase-LTP revealed no difference between Tg and WT mice (Additional file 2: Figure S2). The L-LTP enhancement was age-dependent, as no effect was observed in younger (6 months old) Tg mice compared to WT controls (Additional file 3: Figure S3a). When we divided the mice in three age groups of 6 months, 12 months and 18 months, we found that enhanced L-LTP was detected from 12 months of age (Additional file 3: Figure S3a, 3b and 3c). Because primary neurons are not lost in aged mice of this model [37], we hypothesized that inhibitory GABAergic signaling was impaired in aged Tg mice leading to enhanced potentiation of the hippocampal CA3-CA1 network. To test the idea that GABAergic function was compromised in our pathological tau mouse model, we treated slices with a sub-threshold (no effect on basal synaptic transmission) dose of the GABA_A_ receptor (GABA_A_R) agonist, zolpidem (1 μM) [38]. Zolpidem treatment 10 min prior to stimulation rescued the enhanced L-LTP in hippocampal slices from Tg mice, and importantly, had no effect on L-LTP in WT slices (Figure 2c,d). We also found a significant positive correlation between the potentiated fEPSP slope and the levels of phospho-tau markers PHF1 (53 kD) and CP13 in the cortex (Additional file 4: Figure S4), linking the severity of hippocampal dysfunction to pathological tau expression. These findings support the idea that the exaggerated synaptic plasticity in Tg mice results from reduced GABAergic function, perturbing normal inhibitory-excitatory balance in the CA3-CA1 circuit.

### Loss of hippocampal GABAergic interneurons

**Figure 3:**
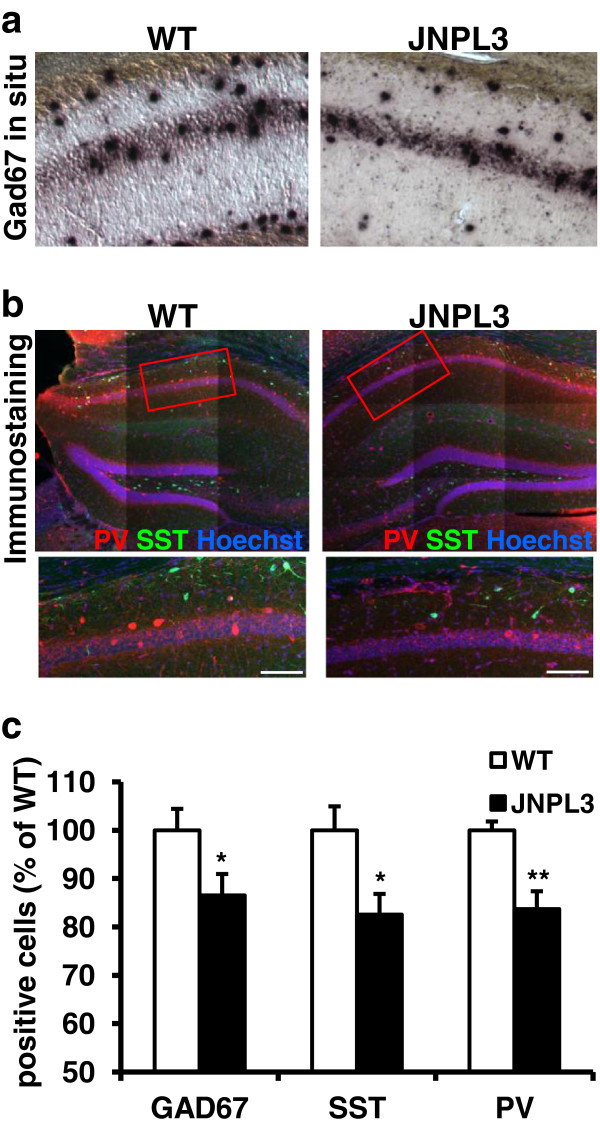
**Loss of GABAergic interneurons in hippocampal area CA1. (a)***In situ* hybridization for the GABAergic marker, GAD67, showed a significant reduction of GAD67-positive neurons in area CA1 (Scale bar = 100 μm). **(b)** Fluorescent immunostaining against parvalbumin (PV, Cy3) and somatostatin (SST, Cy2), counterstained with Hoechst (Upper image are tiled images of 200× magnification, lower panel 400× magnification). **(c)** Tg mice showed significantly reduced numbers of GAD67-positive (WT=9 mice, 69 slices, Tg=8 mice, 60 slices, *t*_15_=2.143, *P=0.0490), SST-positive (WT=6 mice, 61 slices, Tg=7 mice, 61 slices *t*_11_=2.685, *P=0.0212), and PV-positive (WT=7 mice, 61 slices, Tg=7 mice, 61 slices, *t*_12_=3.559, **P=0.0039) neurons in CA1 region in series of brain slices. Data presented as mean + SEM.

One explanation for our results is that JNPL3 (BL6) mice have fewer surface GABA_A_ receptors, reducing neuronal responses to GABA. However, using biotin-surface labeling, we found that total levels and surface levels of several GABA_A_R subunits (α1, β2, and β3) were unchanged in Tg mice compared to WT mice (Additional file 5: Figure S5a, 5b). Another possibility is that hippocampal GABAergic interneurons are reduced or lost in the hippocampus of JNPL3 (BL6) mice. As shown in previous studies, the cell body layer and the stratum oriens in the hippocampus contain GABAergic interneurons that control excitation [39,40]. To test this notion, we used *in situ* hybridization with a GAD67 riboprobe to quantify the number of GABAergic interneurons in the hippocampus. In agreement with this idea, we found significantly fewer (~20%) GAD67-positive cells in hippocampal area CA1 of Tg mice compared to controls (Figure 3a, 3c). Several classes of GABAergic interneurons have been identified using molecularmarkers, including parvalbumin (PV) or somatostatin (SST) positive cells [41]. Because PV- and SST-positive neurons represent the predominant populations of GABAergic interneurons in the hippocampus [42], we next determined whether the GABAergic interneurons reduced in Tg mice are represented by these two different interneuronal types. We found that the number of both PV and SST interneuron subtypes were significantly decreased (~20%) in area CA1 of Tg mice compared to WT mice (Figure 3b, 3c). Additionally, we found significantly fewer SST-positive neurons in the dentate gyrus (DG) (Additional file 6: Figure S6). There was also a trend toward fewer PV-positive cells in the DG, but it was not statistically significant (Additional file 6: Figure S6). The combined results of these experiments indicate that hippocampal GABAergic function in JNPL3 (BL6) mice is compromised as a result of interneuronal cell loss, derived from the most widely represented interneuronal classes in area CA1.

### Pathological tau markers are expressed in GABAergic interneurons

**Figure 4:**
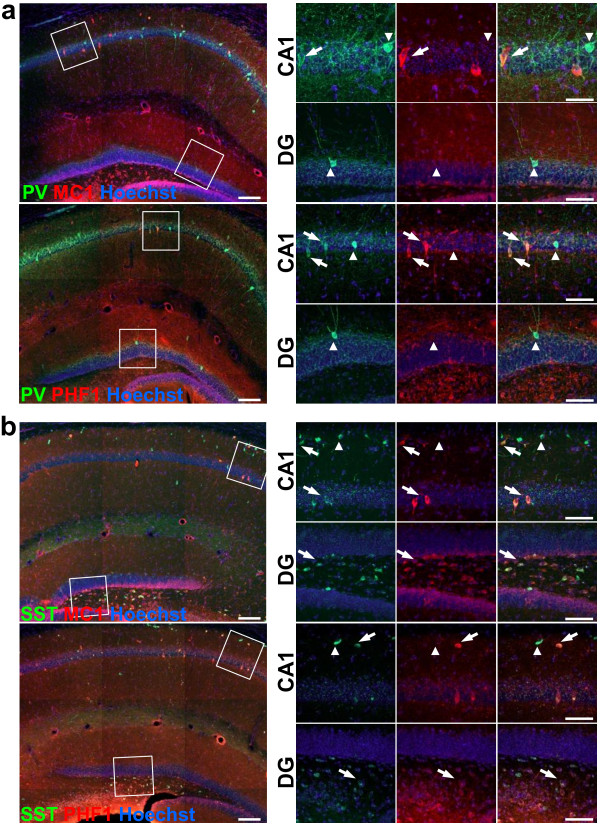
**Colocalization of pathological tau, visualized with MC1 and PHF1 antibodies, in GABAergic interneurons in the hippocampus of aged JNPL3 (BL6) mice. (a)** Fluorescent immunostaining against parvalbumin (PV) and MC1 or PHF1 counterstained with Hoechst shows that PV-positive GABAergic interneurons in the CA1 region express pathological tau. Higher magnification shows that not all neurons express pathological-tau. **(b)** Immunostaining against somatostatin (SST) also shows colocalization with pathological tau with SST-positive GABAergic interneurons. Interestingly, SST strongly colocalizes with pathological-tau in the hilus within the dentate gyrus (left panel; scale bar = 100 μm, right panel; scale bar = 50 μm; arrows show GABAergic interneurons that co-localize with pathological tau markers, while arrowheads show GABAergic interneurons that are not co-localizing with pathological tau markers).

After finding that pathological tau staining is abundantly present and that GABAergic interneurons are reduced in the hippocampus of JNPL3 (BL6) mice we next examined if the pathological tau markers were localized in GABAergic interneurons in aged JNPL3 (BL6) mice. To do this, we perfused mice and stained fixed brain slices for MC1 (an early pathological tau marker) and PV or SST or for PHF1 (a late pathological tau marker) and PV or SST. Strikingly, both GABAergic interneuron subtypes co-localized with MC1 and PHF1 in the hippocampus (Figure 4). We found that a subset of PV-positive neurons in the CA1 region co-localized with MC1 and PHF1 (Figure 4a, arrows). In the DG however, where PV-positive neurons are less abundant, we did not find PV-positive neurons that co-localized with either MC1 or PHF1 (Figure 4a, arrowheads). In contrast, we found that SST-positive interneurons co-localized with MC1 and PHF1 staining in both the CA1 and dentate gyrus (Figure 4b, arrows). These results show that pathological tau markers are present in GABAergic interneurons, which might promote interneuronal loss in the hippocampus.

### Hippocampus-dependent memory and sensorimotor behavioral deficits

**Figure 5:**
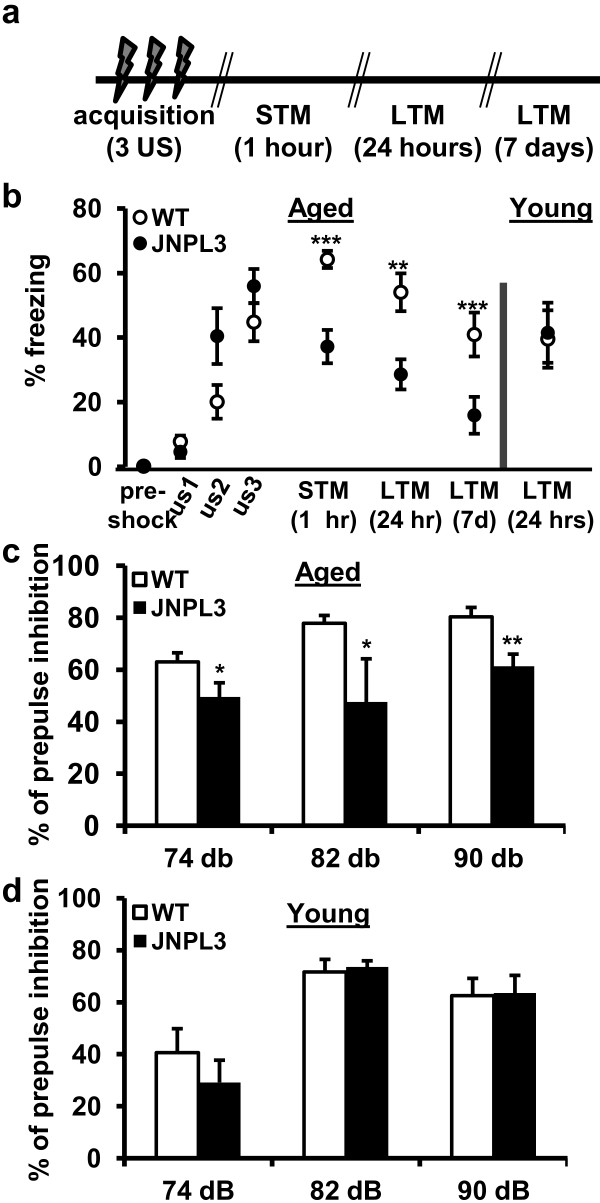
**Over-expression of mutant tau impairs memory and sensori-motor gating in an age dependent fashion (a) Schedule of contextual fear conditioning testing (US=unconditioned stimulus; memory tested at 1h, 24h and 7d delay). (b)** Aged Tg mice (age 12–13 months) showed impaired short-term (STM, 1 h) (WT=11 mice, Tg=12 mice, *t*_21_=4.522, ***P=0.0002) and long-term memory (LTM, 24h: *t*_21_=3.424, **P=0.0026; 7d: *t*_21_=4.283, ***P=0.0003) when compared to WT mice. Young Tg mice (5–6 months) showed normal memory when compared to age-matched WT controls (right panel) (WT=9 mice, Tg=10 mice, *t*_17_=0.2793, *P*=0.7834). **(c)** Sensori-motor gating was impaired in aged Tg mice (WT=17 mice, Tg=8 mice; 72dB: *t*_23_=0.2.130, *P=0.0441; 82dB: *t*_23_=2.532, *P=0.0186, 90dB: *t*_23_=3.040, **P=0.0058). **(d)** Young Tg mice had normal sensorigating when compared to young WT controls (WT=11 mice, Tg=11 mice; 72dB: *t*_20_=0.9115, *P*=0.3729; 82dB: *t*_20_=0.3533, P=0.7276, 90dB: *t*_20_=0.1095, *P*=0.9139). Data are presented as mean ± SEM.; n.s. not significant.

A characteristic feature of tauopathy is dementia, so next we examined memory in aged JNPL3 (BL6) mice. Because we found age-dependent deficits in hippocampal synaptic plasticity in these mice (Additional file 3: Figure S3), we assessed both young and aged JNPL3 (BL6) mice in hippocampus-dependent behavioral tasks. Several hippocampal-dependent behavioral paradigms have been described including the Morris Water Maze or Barnes Maze. However these tasks are highly dependent on locomotor competency. Aged JNPL3 mice in the mixed background develop severe motor deficits [36], and while JNPL3 (BL6) mice are less impaired (data not shown) they do exhibit some locomotor deficits. Therefore, to reduce possible locomotor confounds, we decided to test Tg mice and WT controls using the contextual fear conditioning paradigm, a test that depends on hippocampal function but is less reliant on locomotor performance. Mice were trained by placing them in a training context and exposing them to a brief aversive unconditioned stimulus (US, footshock). Then fear memory assessed by freezing behavior was measured at three time points following training (Figure 5a). Before the administration of the US, no difference was detected in the freezing levels or baseline exploratory locomotor activity between Tg and control mice (Figure 5b). Although aged Tg mice displayed levels of acquisition that was indistinguishable from WT mice, they had severely impaired short-term (1 h) and long-term memory (24 h and 7 days) (Figure 5b). The observed memory deficits were not due to differences in nociception, as aged Tg and WT mice displayed indistinguishable response curves in the hot plate assay (Additional file 7: Figure S7). To determine if the onset of this deficit was age-dependent, we also tested younger mice (5–6 months of age). In contrast to aged Tg mice, young Tg mice showed no contextual fear memory impairment at 24 h (Figure 5b). We also tested prepulse inhibition (PPI) of startle response, a behavioral assay dependent on GABAergic function [43]. Consistent with the loss of the GABAergic interneurons we observed, aged Tg mice had a significantly impaired PPI response compared to WT controls (Figure 5c), whereas young Tg and WT mice did not differ (Figure 5d). These results show that synaptic and behavioral deficits in JNPL3 (BL6) mice are a function of age-related pathological tau expression and may be related to GABAergic dysregulation.

## Discussion

In the current study we examined the effect of mutant tau (P301L) on synaptic plasticity and behavior in aged JNPL3 mice in a C57BL/6 background, a mouse model for tauopathy. We find that aged JNPL3 (BL6) mice show altered hippocampal long-lasting LTP, behavioral abnormalities such as deficits in both short and long term contextual fear memory and reduced sensorimotor gating. Electrophysiological effects in JNPL3 (BL6) mice were corrected by enhancing GABAergic function in the hippocampus. Consistent with a loss of GABAergic tone in Tg mice, we found a reduced number of GABAergic interneurons in area CA1 and dentate gyrus of the hippocampus. Collectively, our data show for the first time that mutant tau has toxic effects on GABAergic interneurons that progresses with age, leading to a loss of GABAergic function, altered hippocampal synaptic plasticity, and impaired memory and sensorigating in aged JNPL3 (BL6) mice.

Aged JNPL3 (BL6) mice show tau pathology consistent with an age-progressive onset of tau pathological markers (Figure 1). Pathology was found in CA1 hippocampal region, especially in the molecular layer containing the dendrites. The tau pathology we detect is, however, less robust compared to JNPL3 mice in a mixed background [36]. This is consistent with previous reports showing that JNPL3 (BL6) mice display a milder pathology than in a mixed background [44]. These findings, combined with ours, establish again that the strain background is an important factor to consider when studying mouse models of human disease. Although the pathology we observe is milder, we think that our study using this C57BL/6 background is very important because it is more comparable to other AD mouse models which use the same genetic background, for example Tau_RD_ (pro and anti-aggregant) [19,45] and hAPPJ20 [46].

We found that expression of P301L tau in aged Tg mice did not impair either basal synaptic transmission or transient forms of synaptic plasticity in the hippocampal Schaffer collateral-area CA1 synaptic circuit. Although other reports have shown alterations in short-term synaptic changes in the hippocampus of AD mouse models [17,47], little was known about long-lasting synaptic changes in aged JNPL3 (BL6) mice. Surprisingly, we found that L-LTP was enhanced in aged JNPL3 (BL6) mice (Figure 2a, Additional file 3: Figure S3). This enhanced L-LTP was rescued with treatment with zolpidem, a GABA_A_-receptor agonist (Figure 2b). These results suggest that GABAergic function is impaired in JNPL3 (BL6) mice. Indeed, supporting this idea, we found that hippocampal GABAergic interneurons in area CA1 were reduced in Tg mice (Figure 3). The L-LTP rescue we observed with zolpidem treatment was not due to over activation of GABA_A_Rs in the CA3-CA1 circuit, as we titrated zolpidem to preclude detectable effects on the L-LTP responses in WT slices. These results, combined with our observations that I/O and PPF were essentially normal in aged JNPL3 (BL6) mice, suggest that although GABAergic interneuronal function is compromised, homeostatic compensation is able, at least partially, to maintain essential circuit integrity in the aged JNPL3 (BL6) hippocampus. Functional deficits may only appear after the synaptic circuit is challenged by activity above ‘nominal’ levels, i.e. in response to behavioral experience or strong stimulation. Future studies, using a more detailed examination of single cell properties may reveal more subtle GABAergic deficits not detectable using field recording approaches. Additionally, it would be interesting to examine if these mice have altered synaptic plasticity in other brain areas, such as the dentate gyrus, where we also found a loss of GABAergic interneurons and a co-localization of pathological tau markers with GABAergic interneurons (Additional file 6: Figure S6 and Additional file 4: Figure S4). Additionally, other AD mouse models have shown GABAergic interneuron and synaptic plasticity deficits in the dentate gyrus [15,17,47,48]. Strikingly, aged JNPL3 (BL6) mice had severe memory deficits and impaired PPI that may be explained by the loss of inhibitory control involved in memory formation and sensorimotor gating. Finding enhanced L-LTP and impaired behavioral performance may seem at first glance to be counterintuitive, but other mouse models have shown enhanced LTP and impaired memory [49-51]. Regardless, these results clearly show that tau plays an important role, separate from Aβ, in synaptic plasticity mechanisms.

Enhanced hippocampal LTP was also reported in very young P301L mice (5–7 weeks of age), but only in the dentate gyrus [17]. This group also showed that young P301L mice had improved performance in the novel object recognition assay. Since no tau pathology was present at this age, the authors suggested that the over-expression of tau resulted in improved trafficking of glutamate receptors, enhancing synaptic transmission and improving memory. Alternative tau-dependent mechanisms may regulate hippocampal synaptic plasticity in an age-dependent fashion. Indeed, this may be likely as we found that aged JNPL3 (BL6) mice with mild but detectable tau pathology showed enhanced L-LTP but also severe memory deficits. Our data show the age-progressive loss of GABAergic interneurons in area CA1, and these data coupled with our zolpidem rescue data support the idea that tau-mediated regulation of GABAergic function is responsible for the enhanced L-LTP we detected. This notion is further supported by the impaired PPI we observe, a behavior that depends on GABAergic function, including in the hippocampus [43,52]. Interestingly, co-staining of pathological-tau antibodies with GABAergic interneuron markers showed extensive co-localization in the hippocampus (Figure 4). This finding indicates that pathological tau is present in GABAergic interneurons. One explanation could be that the prion promoter, which is used to drive the transgene in JNPL3 (BL6), has greater activity in GABAergic interneurons than in pyramidal cells, resulting in greater expression in interneurons. However, this is unlikely because the prion promoter has been shown to be mainly active in the excitatory neurons within the pyramidal layer in the hippocampus [53]. Therefore, it might be that tau pathology in GABAergic interneurons is developed from pathogenic isoforms contributed extracellularly or that GABAergic neurons are particularly susceptible to pathogenic tau. More research is necessary to study the role of GABAergic interneurons in the development of pathology and memory loss in AD.

In AD, it is known that cholinergic and glutamatergic neurotransmission are disrupted, while inhibitory GABAergic neurotransmission, mediated by interneurons, is thought to be well-conserved (reviewed in [54]). Recently however, more evidence is emerging that also GABAergic function is compromised. Limon et al. showed that functional GABA_A_ receptors are lost from the brains of AD patients [30]. Furthermore, during the course of normal aging, hippocampal GABAergic interneurons lose contact boutons [55], while this process is accelerated in hAPP mice (J20 line). An AD mouse model expressing human amyloid precursor protein (hAPP) with Swedish and Indiana mutations [55]. This suggests that the excitation/inhibition balance during aging in hAPP (J20) mice is more severely disrupted. Interestingly, aged hAPP (J20) mice show no loss of GABAergic interneurons. Another AD mouse model, a triple transgenic mouse (TauPS2APP) does show a loss of GABAergic interneurons in the hippocampus of aged mutant mice compared to WT mice [47]. Although this study did not examine persistent forms of LTP in the CA3-CA1 circuit, they found early phase-LTP enhancements in the dentate gyrus of tauPS2APP mice. However, because this mouse model carries three transgenes, it was not clear whether all or one mutation contributed to the loss of GABAergic interneurons. Our results suggest that expression of mutant tau protein alone likely promotes the loss of hippocampal interneurons. Furthermore, several reports provide evidence that the development of AD leads to hyperexcitability, shown by the increased incidence of epileptic activity in sporadic AD, which is particularly high in early-onset autosomal-dominant AD [20,21,56,57]. One possible explanation for this may be that GABAergic interneuronal function, which is critical for maintaining excitatory/inhibitory balance in the brain, is also impacted in tauopathies like AD. AD mouse models provide experimental support for this model of AD-related hyperexcitability. For example, the hAPP (J20) mice, was shown to have spontaneous epileptic activity, indicating network hypersynchrony [46,55]. Interestingly, this network hypersynchrony in the hAPPJ20 model resulted from PV cell dysfunction. Furthermore, in an earlier study the same research group showed that reduction of tau in the hAPP (J20) mouse model prevented behavioral deficits and excitotoxicity [14]. Finally, the apoE4 knock-in (KI) mouse model has impaired neurogenesis in the dentate gyrus that was due to impaired presynaptic GABAergic input and resulted in loss of GABAergic interneurons [15,48]. These mice also showed spatial learning and memory deficits, which could be rescued with treatment with the GABA_A_R potentiator pentobarbital [15]. Interestingly, the GABAergic impairment was dependent on tau because apoE4 KI mice in a tau knockout background did not exhibit this phenotype [15]. These results support the idea that tau plays an important role in the survival and function of GABAergic interneurons. To note, these findings highlight the importance of tau in AD-related phenotypes but do not address whether tau lesions on their own manifest effects in AD-related synaptic plasticity and hyperexcitability. Our present findings provide evidence supporting a model directly implicating tau function in the maintenance of balanced neuronal signaling networks.

Two other tau mouse models, which express either an anti- or a pro-aggregant tau protein have been studied to examine the effects of tau aggregation on synaptic plasticity and behavior. While these models are not represented by naturally occurring human mutations, they do provide insight into the role of tau in synaptic plasticity. Pro-aggregant tau resulted in impaired L-LTP in the CA3-CA1 hippocampal pathway, while an anti-aggregant tau model showed enhanced CA1-LTP [19,58]. However, only the pro-aggregant mice showed memory deficits, while the anti-aggregant displayed normal memory, as assessed by Morris water maze and the passive avoidance task. The Tg mice in our study exhibit severe memory deficits and enhanced L-LTP and express a mutant tau isoform with properties that more closely align with pro-aggregant tau. While differences in the effects on LTP between this study and our own may be explained by the different models used, another intriguing possibility is that greater levels of tau aggregation may affect other neuronal signaling pathways involved in the expression of L-LTP that are not impacted by the less severe pathology present in our model. This finding also supports the idea that tau conformation can have different effects on synaptic function and memory, and tau mutations that promote pathological aggregation may impair hippocampal function. This possibility may be useful to consider for developing therapeutic strategies that target tau aggregation.

In summary, this study provides valuable new evidence for a role of tau independent of Aβ in AD-related synaptic deficits. Our data support a model in which tau helps to maintain proper network excitability through the regulation of GABAergic function. ‘Normal’ cognition requires intact neuromolecular pathways for the regulation of synaptic plasticity. Altered synaptic function and memory deficits are major hallmarks of dementia. Results from this study suggest a promising therapeutic avenue for the treatment of AD and FTD may be developing new drug regimens based on removing existing or inhibiting the development of tau pathology. However, effective application of this strategy requires detailed knowledge about the effects of tau pathology on cognition in preclinical model systems. By identifying GABAergic function as a crucial pathway influenced by the expression of pathological tau, we may in the short-term be able to take advantage of several FDA approved drugs regulating GABAergic function for use in AD treatment. In the long-term, these studies may provide new insight into the neuronal signaling pathways and molecular targets that are regulated by tau to develop more effective approaches for tauopathy treatment.

## Competing interests

The authors declare that they have no competing interests.

## Authors' contributions

JL carried out the electrophysiological studies, participated in fear conditioning behavior experiments, histological analyses, performed surface labeling experiments and drafted the manuscript. PK and HW carried out the immunoblotting and edited the manuscript. HR sectioned fixed tissues, performed immunostaining, and assisted with editing the manuscript. HW contributed significantly to the discussion. PC performed PPI experiments, analyzed data, and assisted with the statistical analyses. TF participated in the design of the study assisted with the drafting and editing of the manuscript. CH conceived of the study, analyzed fear conditioning experiments and performed statistical analyses. CH and ES participated in its design and coordination and helped to draft and edit the manuscript. All authors read and approved the final manuscript.

## Supplementary Material

Additional file 1: Figure S1 MC1 and PHF1 staining in the hippocampus of WT mice. No specific MC1 or PHF1 staining is present in the hippocampus of aged WT mice. Because the brains were used simultaneously for *ex vivo* slice electrophysiology, this hemisphere was fixed without perfusing and blood vessel staining is visible. Inset is higher magnification of the highlighted area. (DG=dentate gyrus, CA1= hippocampal area CA1; scale bar = 200 μm). Click here for file

Additional file 2: Figure S2 Basal synaptic transmission, paired-pulse facilitation and E-LTP are not impaired in JNPL3 (BL6) mice. (a) Input versus output plot indicates that aged (>12 month old) Tg and WT mice have comparable fEPSP slopes evoked by increasing stimulation (WT = 21 slices, Tg = 20 slices, *F*_(1,39)_=0.703, *p*=0.407). (b) Tg mice exhibit normal PPF compared to WT mice. The percent facilitation, determined by the ratio of the second fEPSP to the second fEPSP, is shown as interpulse intervals from 10-300ms (WT= 6 mice, 20 slices, Tg=6 mice, 20 slices, *F*_(1,38)_=0.384, *p*=0.539). (c) A single train of HFS evoked similar levels of -E-LTP in Tg and WT mice that decayed to baseline after 80 minutes (WT= 3 mice, 5 slices; Tg= 3 mice, 6 slices, *F*_(1,9)_=0.369, *p*=0.559). Click here for file

Additional file 3: Figure S3 Altered synaptic plasticity in JNPL3 (BL6) mice is age dependent. (a) No difference in L-LTP between young JNPL3 (BL6) and WT mice (age 6–7 months) (WT= 5 mice, 8 slices, Tg=4 mice, 9 slices, *F*_(1,15)_=0.129, *p=*0.725). (b) L-LTP is found to be enhanced in JNPL3 (BL6) mice at 12 months (WT=3 mice, 10 slices; Tg=3 mice, 9 slices; *F*_(1,17)_=7.384, *p*=0.015), and (c) at 18 months (WT=6 mice, 14 slices, Tg=9 mice, 19 slices, *F*_*(1*_*-*_31)_=2.193, *p=0.0359). Click here for file

Additional file 4: Figure S4 fEPSP correlates with levels of pathological tau. The level of pathological tau of the mice used for electrophysiology was plotted against the fEPSP slope at 180 min after HFS induction. There is a correlation between the amount of pathological tau levels and the enhanced fEPSP slope suggesting a link between the amount of pathological tau and altered synaptic plasticity. Click here for file

Additional file 5: Figure S5 Surface labeling of JNPL3 (BL6) and WT mice show no difference in the levels of GABA_A_R_α1_ or GABA_A_R_ß2,3_ receptors (a) No differences in levels of GABA_A_R_α1_, GABA_A_R_ß2,3_ or synaptophysin are found in the total hippocampal lysates derived from aged Tg mice compared to WT mice (n=4 each). (b) Immunoprecipitation of biotin-labeled surface proteins show no differences in number of surface GABA_α1_ receptor in aged Tg mice compared to WT mice. GAPDH is also present on the extracellular matrix [59] and can therefore be used as a loading control (n=4 each). Click here for file

Additional file 6: Figure S6 Reduction of somatostatin (SST)-positive GABAergic interneurons in the dentate gyrus (DG). Number of SST-positive GABAergic interneurons is reduced in the DG of JNPL3 (BL6) mice. Number of PV-positive GABAergic interneurons in the DG is also reduced, although not statistically significant (SST; WT=6 mice, 54 slices, Tg=7 mice, 59 slices *t*_11_=2.216, *P=0.0487 and PV; WT=7 mice, 54 slices, Tg=7 mice, 59 slices, *t*_12_=1.916, *P*=0.0776). Click here for file

Additional file 7: Figure S7 Nociception is not different in aged JNPL3 (BL6) compared to WT animals (age 13–14 months). To determine if there is any difference in nociception between Tg and WT mice, we tested their reflex on a hotplate preheated to 50°C. No difference in nociception was found, suggesting that JNPL3 (BL6) are able to sense the foot shocks given during the contextual fear memory test equally as well as WT mice (WT=5 mice, Tg= 6 mice, *t*_9_=0.6690, *p=*0.52). Click here for file
