# Supplementary figures and images for: Tau pathology induces loss of GABAergic interneurons leading to altered synaptic plasticity and behavioral impairments
Source: Acta Neuropathol Commun. 2013 Jul 11;1:34. doi: 10.1186/2051-5960-1-34 (PMC3893396; doi:10.1186/2051-5960-1-34)

## Supplemental Figure 1

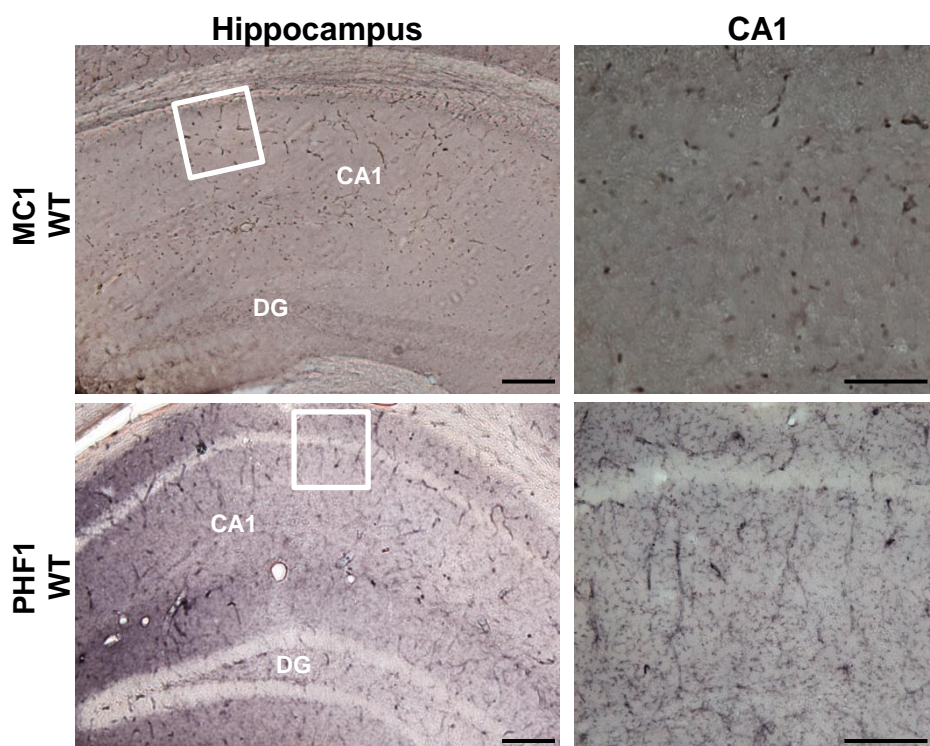

Supplement: Additional file 1: Figure S1 — MC1 and PHF1 staining in the hippocampus of WT mice. No specific MC1 or PHF1 staining is present in the hippocampus of aged WT mice. Because the brains were used simultaneously for ex vivo slice electrophysiology, this hemisphere was fixed without perfusing and blood vessel staining is visible. Inset is higher magnification of the highlighted area. (DG=dentate gyrus, CA1= hippocampal area CA1; scale bar = 200 μm). [file 2051-5960-1-34-S1.pdf]

## Supplemental Figure 2

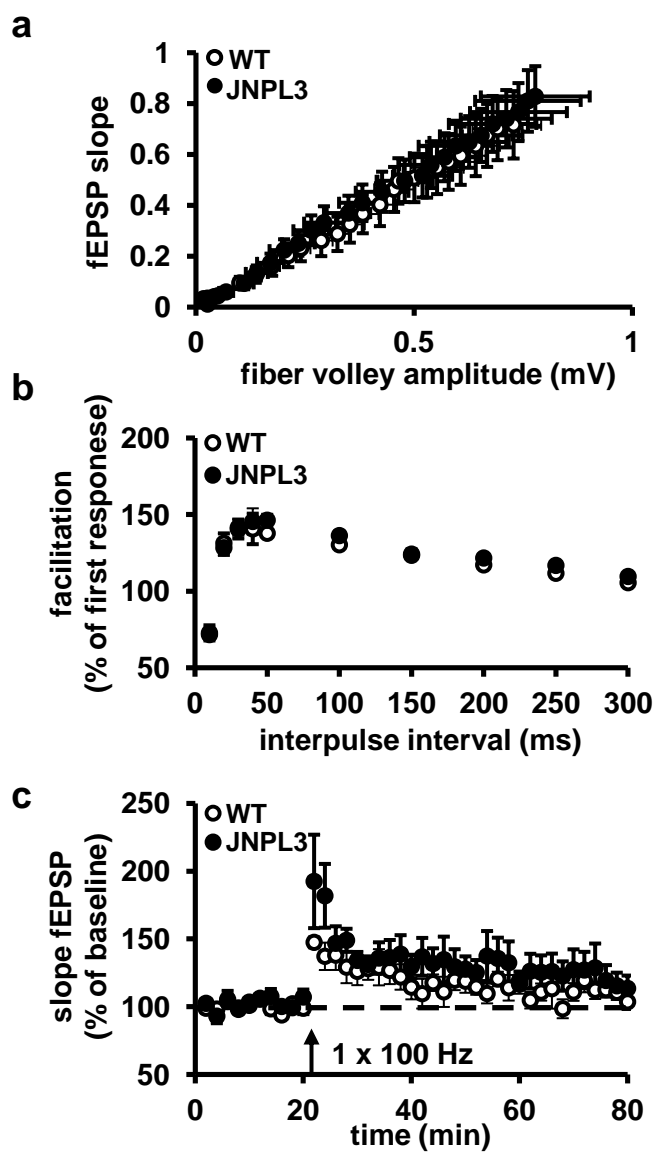

Supplement: Additional file 2: Figure S2 — Basal synaptic transmission, paired-pulse facilitation and E-LTP are not impaired in JNPL3 (BL6) mice. (a) Input versus output plot indicates that aged (>12 month old) Tg and WT mice have comparable fEPSP slopes evoked by increasing stimulation (WT = 21 slices, Tg = 20 slices, F(1,39)=0.703, p=0.407). (b) Tg mice exhibit normal PPF compared to WT mice. The percent facilitation, determined by the ratio of the second fEPSP to the second fEPSP, is shown as interpulse intervals from 10-300ms (WT= 6 mice, 20 slices, Tg=6 mice, 20 slices, F(1,38)=0.384, p=0.539). (c) A single train of HFS evoked similar levels of -E-LTP in Tg and WT mice that decayed to baseline after 80 minutes (WT= 3 mice, 5 slices; Tg= 3 mice, 6 slices, F(1,9)=0.369, p=0.559). [file 2051-5960-1-34-S2.pdf]

### Supplemental figure 3

a

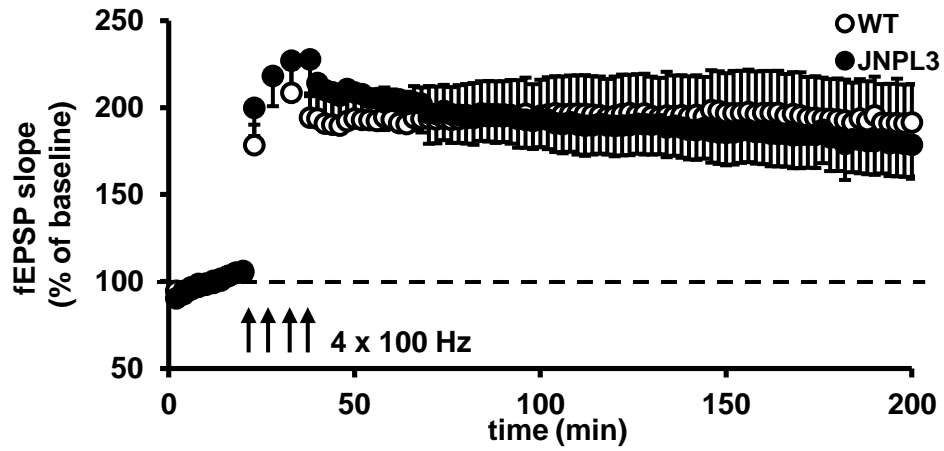

b

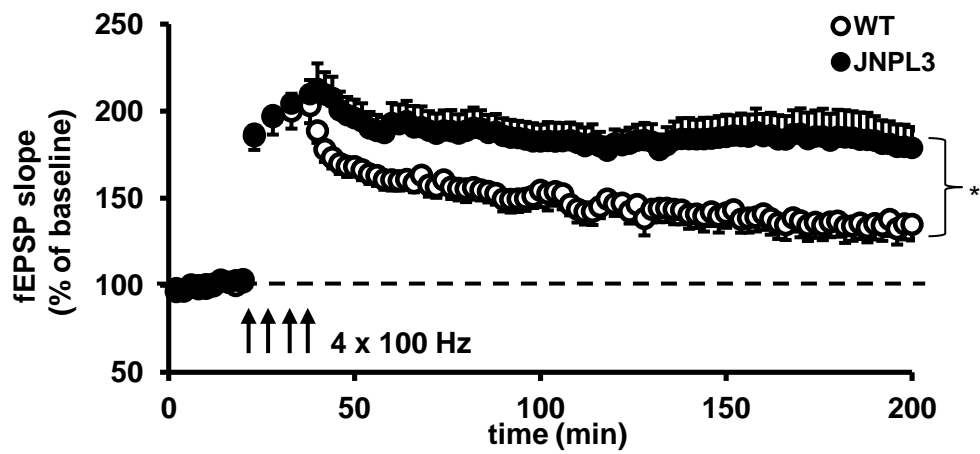

c

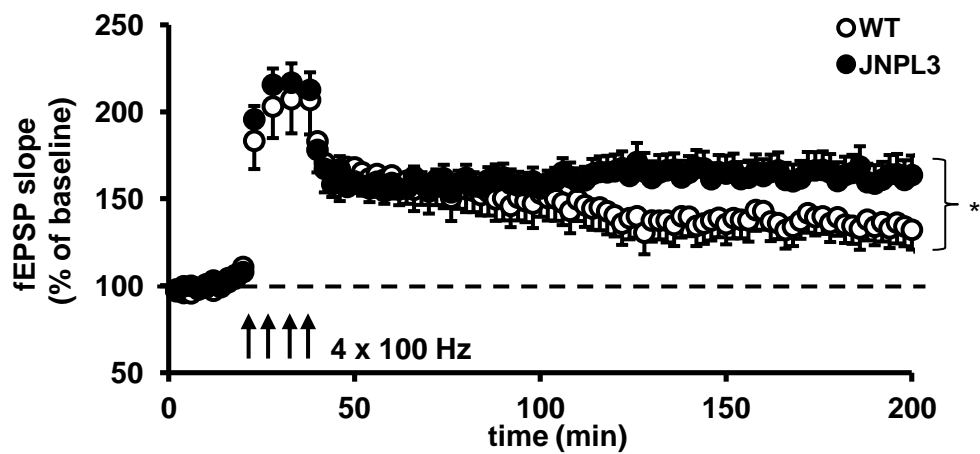

Supplement: Additional file 3: Figure S3 — Altered synaptic plasticity in JNPL3 (BL6) mice is age dependent. (a) No difference in L-LTP between young JNPL3 (BL6) and WT mice (age 6–7 months) (WT= 5 mice, 8 slices, Tg=4 mice, 9 slices, F(1,15)=0.129, p=0.725). (b) L-LTP is found to be enhanced in JNPL3 (BL6) mice at 12 months (WT=3 mice, 10 slices; Tg=3 mice, 9 slices; F(1,17)=7.384, p=0.015), and (c) at 18 months (WT=6 mice, 14 slices, Tg=9 mice, 19 slices, F(1-31)=2.193, *p=0.0359). [file 2051-5960-1-34-S3.pdf]

# Supplemental Figure 4

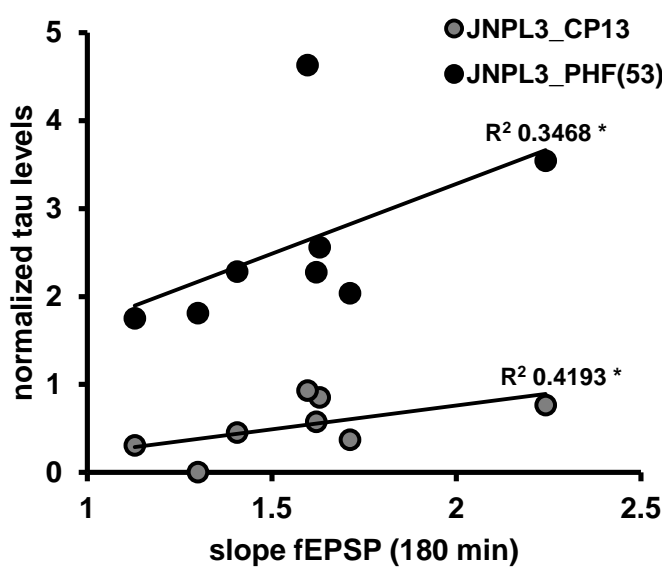

Supplement: Additional file 4: Figure S4 — fEPSP correlates with levels of pathological tau. The level of pathological tau of the mice used for electrophysiology was plotted against the fEPSP slope at 180 min after HFS induction. There is a correlation between the amount of pathological tau levels and the enhanced fEPSP slope suggesting a link between the amount of pathological tau and altered synaptic plasticity. [file 2051-5960-1-34-S4.pdf]

## Supplemental Figure 5

**a**

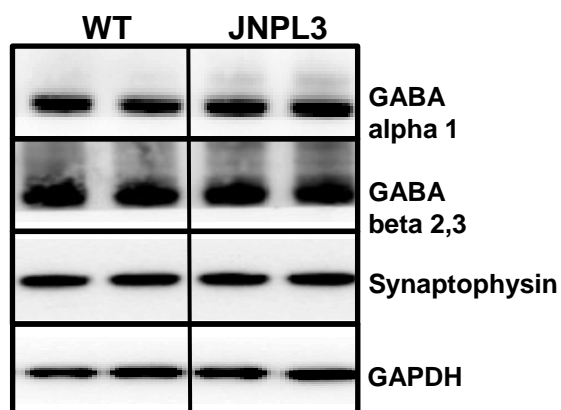

**b**

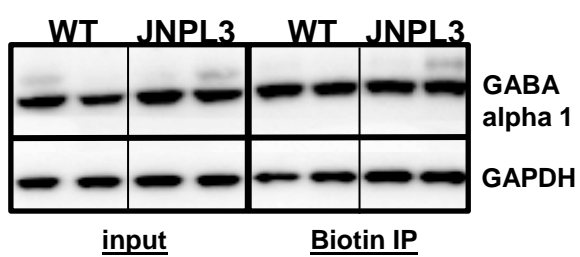

Supplement: Additional file 5: Figure S5 — Surface labeling of JNPL3 (BL6) and WT mice show no difference in the levels of GABAARα1 or GABAARß2,3 receptors (a) No differences in levels of GABAARα1, GABAARß2,3 or synaptophysin are found in the total hippocampal lysates derived from aged Tg mice compared to WT mice (n=4 each). (b) Immunoprecipitation of biotin-labeled surface proteins show no differences in number of surface GABAα1 receptor in aged Tg mice compared to WT mice. GAPDH is also present on the extracellular matrix [59] and can therefore be used as a loading control (n=4 each). [file 2051-5960-1-34-S5.pdf]

## Supplemental Figure 6

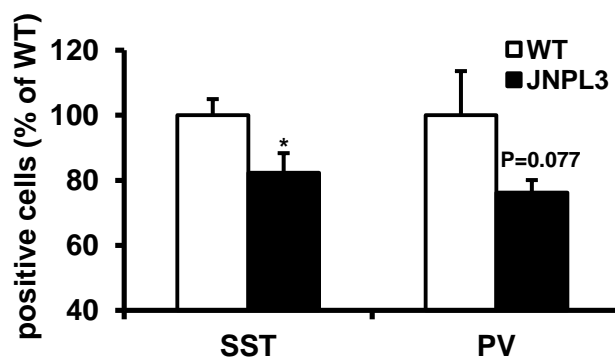

Supplement: Additional file 6: Figure S6 — Reduction of somatostatin (SST)-positive GABAergic interneurons in the dentate gyrus (DG). Number of SST-positive GABAergic interneurons is reduced in the DG of JNPL3 (BL6) mice. Number of PV-positive GABAergic interneurons in the DG is also reduced, although not statistically significant (SST; WT=6 mice, 54 slices, Tg=7 mice, 59 slices t11=2.216, *P=0.0487 and PV; WT=7 mice, 54 slices, Tg=7 mice, 59 slices, t12=1.916, P=0.0776). [file 2051-5960-1-34-S6.pdf]

## Supplemental Figure 7

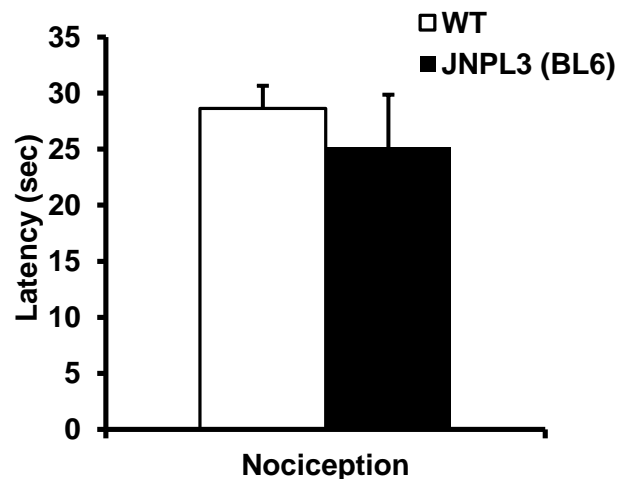

Supplement: Additional file 7: Figure S7 — Nociception is not different in aged JNPL3 (BL6) compared to WT animals (age 13–14 months). To determine if there is any difference in nociception between Tg and WT mice, we tested their reflex on a hotplate preheated to 50°C. No difference in nociception was found, suggesting that JNPL3 (BL6) are able to sense the foot shocks given during the contextual fear memory test equally as well as WT mice (WT=5 mice, Tg= 6 mice, t9=0.6690, p=0.52). [file 2051-5960-1-34-S7.pdf]
